# Comparative evaluation of image quality from three CT simulation scanners

**DOI:** 10.1120/jacmp.v5i4.1978

**Published:** 2004-11-24

**Authors:** Claire McCann, Hamideh Alasti

**Affiliations:** ^1^ Department of Medical Biophysics University of Toronto 610 University Avenue Toronto Ontario Canada; ^2^ Department of Radiation Physics Princess Margaret Hospital 610 University Avenue Toronto Ontario M5G 2M9; ^3^ Department of Radiation Oncology Faculty of Medicine, University of Toronto Canada

**Keywords:** CT simulator, single‐slice CT, multislice CT, image quality

## Abstract

Today, radiation therapy (RT) is moving toward increased radiation dose to the tumor as a result of 3D conformal RT (3DCRT) and intensity‐modulated RT (IMRT), which have been made possible by advances in volumetric‐based image planning with digital imaging systems such as computed tomography (CT). Treatment planning for such RT requires superior CT image quality. Our goal in this study was to evaluate and to compare the image quality of three unique CT simulation scanners available at our center for both single‐ and multiple‐slice helical scanners. These scanners included a conventional 70‐cm bore single‐slice scanner (Philips Medical Systems), a large 85‐cm bore single‐slice scanner (Philips Medical Systems), and a 70‐cm bore multislice scanner (GE Medical Systems). Image quality was evaluated in terms of image noise, low‐contrast detectability (LCD), limiting spatial resolution (modulation transfer function), and slice thickness accuracy in accordance with guidelines set out by the AAPM. A commercially available Catphan® phantom was used to characterize image quality for both axial and helical modes of scanning. We found that image quality was generally comparable for all scanners. Limiting spatial resolution and slice thickness accuracy were comparable for all three scanners for both scanning modes. The multislice unit was superior in terms of noise content, resulting in improved visualization of small, low‐contrast objects, which is of significant clinical importance, particularly for soft tissue delineation. In addition, the multislice unit optimizes volume coverage speed and longitudinal resolution without compromising image quality, a significant advantage for the radiation oncology environment.

PACS numbers: 87.57.Ce, 87.59.Fm, 87.57.Nk

## I. INTRODUCTION

The goal of radiation therapy (RT) is to deliver a therapeutic dose of radiation to the tumor without damaging surrounding healthy tissues. Delivery of a curable radiation dose is limited, however, by normal tissue tolerance, necessitating the move toward more precise 3D dose delivery with techniques such as 3D conformal radiation therapy (3DCRT) or intensity‐modulated RT (IMRT). These techniques provide a more effective means of achieving improved tumor coverage with a therapeutic dose of radiation while maintaining normal tissue complications at a minimum. These improvements in RT have been made possible by advances in volumetric‐based image planning with digital imaging systems such as computed tomography (CT), together with the standardization of guidelines for defining tumor volume.^(^ ^1^ ^)^

CT for radiotherapy planning or CT simulation involves the acquisition of a CT image data set of the patient and the subsequent treatment simulation using sophisticated virtual beam displays in three dimensions based on digitally reconstructed radiographs. CT simulation systems can reconstruct images in any plane and can generate volume‐rendered displays that allow for improved perspective of tumors and normal tissue, necessary for the delineation of the tighter margins around the clinical target volume as defined by 3DCRT and IMRT. Due to the escalation in radiation dose permitted by these techniques, superior CT image quality is critical for accurate delineation of the tumor volume and critical tissue structures to ensure complete tumor destruction and healthy tissue preservation.

The importance of superior image quality required of these volumetric‐based, escalated‐dose radiotherapy techniques motivated us to characterize and evaluate the image quality of the CT simulation scanners available at our institution. Our center, Princess Margaret Hospital in Toronto, Canada, is equipped with three CT simulation systems: a 70‐cm bore size multislice scanner (LightSpeed Plus, General Electric Medical Systems, Chicago, IL), a 70‐cm bore single‐slice scanner (UltraZ, Philips Medical Systems Inc., Cleveland, OH), and an 85‐cm bore single‐slice scanner (AcQSim, Philips Medical Systems, Inc., Cleveland, OH). While other scanners do exist, these three systems are reasonably representative of the different CT scanners in the RT planning environment today. We have optimized the use of each scanner for clinical applications suited to the characteristics of each particular unit. The 70‐cm bore single‐slice Philips scanner was the first unit introduced into our radiation oncology clinic and is generally used for scanning the prostate as well as head and neck regions. The 85‐cm large‐bore single‐slice Philips scanner was later introduced to accommodate larger patients and those requiring special immobilization or large setup devices. This unit is generally used for scanning breast and mantel regions, as well as gynecological areas.

The 70‐cm bore multislice GE scanner is the most recent addition to the radiation oncology department at Princess Margaret Hospital and is used for scanning pediatric patients as well as thoracic, abdominal, and gastrointestinal regions. This unit is equipped with a multiple‐row detector array, which can collect data at different longitudinal (*z*) locations simultaneously, as opposed to a single‐row detector array characteristic of conventional single‐slice CT scanners.^(^ ^2^ ^,^ ^3^ ^)^ A significant advantage of multislice scanning is the ability to achieve high‐volume coverage speed and high‐longitudinal (z‐axis) resolution simultaneously, limiting the scan time and radiation dose to the patient while achieving high‐quality images. According to Hu,^(^ ^2^ ^)^ volume‐coverage speed is defined as rapidly scanning a large (*z*) volume with high‐longitudinal resolution and low‐image artifacts. Single‐slice scanners must sacrifice one for the other because the increased X‐ray collimation required for faster volume coverage compromises resolution in the longitudinal direction. Multiple rows of detectors allow the subdivision of a large X‐ray beam (required for fast volume coverage) into multiple subdivided beams (essential for reasonable *z*‐axis resolution).

Image quality in both single‐ and multislice helical scanning systems is dictated in part by a parameter known as pitch. Traditionally, in the single‐slice system, helical pitch is defined as the ratio of the table advancement per rotation (mm/rot) to the X‐ray beam collimation,^(^ ^2^ ^,^ ^3^ ^)^ which is the width of the incoming X‐ray beam along the *z*‐axis, or scan width. In the multislice case, helical pitch has been defined by some as the ratio of the table advancement per rotation (mm/rot) to the z‐axis width (*t*) of *one* channel of an *N*‐channel detector,^(^ ^2^ ^,^ ^3^ ^)^ where the channel itself can have different widths depending on the detector configuration selected by the operator. In order to maintain the established and intuitive meaning of pitch, McCollough^(^ ^3^ ^)^ and the International Electrotechnical Committee Standard (IECS)^(^ ^4^ ^)^ have defined a single, inclusive definition of pitch to accommodate both single‐ and multislice scanners. In this definition, pitch is equal to the ratio of the table travel advancement per rotation to the total nominal scan width (irradiated width) along the z‐axis. For the single‐slice scanner, total nominal scan width is defined as the X‐ray beam collimation. For the multislice scanner, total nominal scan width is defined as the *total* number of active detector channels (*N*) multiplied by the *z*‐axis width (*t*) of single channel (where *t* depends on the detector configuration selected and on the width of a single detector element). With this comprehensive definition, the relationship between dose and pitch is maintained, that is, dose is inversely proportional to pitch.^(^ ^3^ ^)^ There are two helical pitch selections offered by the manufacturers of this scanner, which are identified as high quality (HQ) and high speed (HS) for pitches of 3:1 and 6:1, respectively. General Electric (GE) has defined these pitch values according to the noninclusive definition of pitch, which is the ratio of table advancement per rotation (mm/rot) to the z‐axis width of *one* channel of an *N*‐channel detector.^(^ ^2^ ^)^ According to the IECS definition of pitch, then, for a multislice scanner (ratio of the table travel advancement per rotation (mm/rot) to the total nominal scan width), a GE‐defined pitch of 3:1 corresponds to an IECS pitch of 0.75. This is consistent with a 25% overlap of the radiation beam between consecutive rotations. Similarly, a GE‐defined pitch of 6:1 corresponds to an IECS pitch of 1.5, resulting in a gap along the *z*‐axis, requiring interpolation between adjacent slices for reconstruction of data in the region of the gap. In theory, the HQ mode yields better image quality, particularly good contrast resolution and low‐image artifacts, while the HS mode is provided for those applications demanding high‐volume coverage speed and higher table speed, compromising image quality.

The purpose of this study is to evaluate and to make direct comparisons between the image quality performance of three uniquely different scanners: a 70‐cm bore multislice GE scanner and 70‐cm and 85‐cm bore single‐slice Philips scanners, which to our knowledge has not been done. According to AAPM Nos. 39 and 74, quality control of CT image quality includes the evaluation of image slice thickness (or slice sensitivity profile), spatial resolution (image sharpness), low‐contrast detectability (LCD), and image noise.^(^ ^5^ ^,^ ^6^ ^)^ In this study, image quality was defined in terms of the aforementioned characteristics. We have analyzed and compared the effect of varying user‐controlled imaging parameters on the quality of CT images generated by three unique CT systems used for RT planning. Effective and safe RT depends on the ability to accurately target the tumor and surrounding tissues, which in turn depends on the quality of the images. Superior image quality will improve the visualization and delineation of the tumor volume and healthy tissue structures. Another important aspect of quality control for CT scanners is dose. Although this parameter dose not provide information on image quality directly, dosimetry of single‐slice scans (CT dose index, CTDI) is important for the determination of radiation dose delivered to the patient. CTDI was evaluated in separate phantoms and experiments and is included at the end of this report.

## II. MATERIALS

The LightSpeed Plus scanner (GE Medical Systems, Inc., Chicago, IL) is a third‐generation (the tube and the detector array are attached to the same rotating frame and move simultaneously) multislice scanner, with a 70‐cm patient aperture and acquires four data sets every 0.5 s. Thus the scanner is equipped with four output channels and a matrix detector built on GE‐patented HiLight polycrystalline ceramic technology that consists of a total of sixteen 1.25‐mm detector elements (or cells) along the *z*‐axis.^(^ ^7^ ^)^ Depending on the desired channel thickness, the 16 detector cells can be summed to provide a “macro‐cell,” resulting in a channel width (*t*) or detector row collimation of 1.25 mm (one detector element, no summation), 2.5 mm (two detector elements), 3.75 mm (three detector elements), or 5 mm (four detector elements). In other words, four images can be acquired simultaneously with a minimum detector row collimation of 1.25 mm (resulting in a 5‐mm total nominal scan width), or a maximum detector row collimation of 5 mm (resulting in a 20‐mm total nominal scan width). Other scanner features include a 541‐mm distance from the focal spot to the imaging isocenter, as opposed to a distance of 630 mm, characteristic of most single‐slice scanners. This “shorter” geometry provides better X‐ray intensity and reduces the centrifugal force on the tube, allowing for faster rotational speeds. This multislice scanner consists of a rotating graphite‐based target with a maximum heat capacity of $6.30\times 10^{6}$ HU/4700 kJ (Hounsfield units, HU). It has a maximum and minimum scanning field of view (SFOV) of 496.9 mm and 250 mm, respectively, and a user‐adjustable display field of view (DFOV) ranging from 96 mm to 500 mm. This scanner is equipped with a large and small focal spot with dimensions 1.2mm×1.2mm and 0.9mm×0.7mm, respectively. As mentioned previously, the LightSpeed Plus scanner offers two helical scanning modes, HQ and HS, which correspond to GE pitches of 3 and 6, respectively, and IECS pitches of 0.75 and 1.5, respectively.

Another useful feature of this scanner is the ability to reconstruct images postscanning with variable image thicknesses, made possible with helical mode scanning. Image thicknesses greater than or equal to the macro‐cell channel width can be reconstructed. Four X‐ray tube potential settings are available to specify desired X‐ray energy intensity (80 kVp, 100 kVp, 120 kVp, and 140 kVp). Exposure techniques range from 10 mA to 440 mA adjustable by 10‐mA increments with nine scan‐time settings of 0.5 s, 0.6 s, 0.7 s, 0.8 s, 0.9 s, 1.0 s, 2.0 s, 3.0 s, and 4.0 s for axial scan selections and a maximum helical scanning time of 120 s for a single continuous exposure. This scanner will be referred to as the 70‐cm bore multislice unit.

The AcQSim scanner (Philips Medical Systems, Inc., Cleveland, OH) is a fourth‐generation single‐slice scanner (X‐ray tube rotates about fixed detectors positioned 360°), with an 85‐cm patient aperture. The large‐bore scanner has a ring of individual cadmium‐tungstate detectors. The detector elements are divided into 120 detector modules, each module containing two 10‐element crystal arrays with a detector center‐to‐center distance of 2.2 mm.^(^ ^8^ ^)^ The distance from the focal spot to the imaging isocenter is 651 mm. This scanner consists of a rotating graphite‐based target with a maximum heat capacity of $6.50\times 10^{6}$ HU and a manufacturer‐specified tube cooling rate of 650 times 10^3^ HU/min. This large‐bore scanner has a single constant SFOV of 600 mm and a user‐adjustable DFOV ranging from 40 mm to 600 mm. This scanner is equipped with large and small focal spot sizes with dimensions 0.6mm×0.9 and 0.4mm×0.5mm, respectively, and offers helical pitch selections of 0.5 to 3. Nominal slice thicknesses of 2 mm, 3 mm, 4 mm, 5 mm, and 8 mm are available. In addition, five settings are available to specify desired voltage potentials (80 kVp, 100 kVp, 120 kVp, 130 kVp, and 140 kVp). Exposure techniques range from 30 mA to 400 mA, adjustable by 10‐mA increments with five scan‐time settings of 1.0 s, 1.5 s, 2.0 s, 3.0 s, and 4.0 s for axial mode scan selections. This scanner will be referred to as the 85‐cm bore single‐slice unit. The 70‐cm bore UltraZ scanner (Philips Medical Systems, Inc., Cleveland, OH) is a single‐slice scanner with a design similar to that of the 85‐cm bore scanner. Unlike the large bore, however, the UltraZ scanner has a variable, although limited, SFOV ranging from 250 mm to 480 mm and a display range of 40 mm to 480 mm (DFOV).^(^ ^9^ ^)^ It is also equipped with two focal spot sizes of 0.6mm×0.9 and 0.4mm×0.5mm, respectively. This scanner will be referred to as the 70‐cm bore single‐slice unit. It is worth noting that Philips also makes a 4800‐element detector, but our clinic was not equipped with this scanner for testing and comparing image quality.

Comparable sets of image quality measurements were performed on all three scanners using the commercially available CT performance phantom, Catphan® 500 (The Phantom Laboratory, Inc., Salem, NY). The modular design of the phantom enables the assessment of different image quality parameters independent of one another. The phantom is designed so that all modules can be located by precisely indexing the table from the center of the first module (section CTP 401) to the center of each subsequent test module.^(^ ^10^ ^)^ Four image quality parameters were evaluated using the Catphan® phantom. The solid image uniformity module (CTP486) was used to evaluate image noise. Spatial frequency limits were calculated based on the modulation transfer function (MTF) determined with the use of a point source, a tungsten carbide bead (0.28 mm diameter) contained in module CTP528. LCD was evaluated using module CTP515, which contains supra‐slice targets with nominal contrast levels of 1.0%, 0.5%, and 0.3%. Verification of slice thickness was determined with two pairs of 23° wire ramps contained in the CTP401 module. The phantom was always positioned at the isocenter of gantry.

## III. METHODS

Image quality for each scanner was evaluated in terms of image noise, MTF, LCD, and slice thickness accuracy for a variety of user‐specified imaging parameters. Methods for determination of these image quality characteristics were based on those described in the Catphan® manual.^(^ ^10^ ^)^ For comparative purposes, the same or similar scan parameters available on each scanner were selected. User‐adjusted scan parameters included milliamperes, slice thickness, and SFOV. Image quality was evaluated for two scanning modes: axial and helical. Axial scan parameters were selected to reflect a wide range of scanning protocols to test the performance of each scanner at either end of the scanning spectrum (see Tables 1 and 2). Single‐slice helical mode image quality comparisons were made between the 85‐cm bore single‐slice and the 70‐bore multislice units for a large SFOV only because the large bore is limited to a 600‐mm SFOV. For a valid comparison, the maximum SFOV available on the 70‐cm bore multislice unit (500 mm) was selected. Due to specific scanning restrictions imposed by the 70‐cm bore single‐slice unit, axial mode image quality for this scanner was compared to the image quality of the 70‐cm bore multislice unit at a small SFOV (250 mm) only. This is the smallest SFOV available on both scanners. The image quality of each scanner was also evaluated and compared for helical mode scanning, which is the scanning mode most often used for RT planning. Helical scanning mode parameters were selected to reflect established clinical protocols, including the pelvis (protocol I), mantle (protocol II), and whole central nervous system (protocol III). Selection of identical scan parameters for each scanner was not always possible due to the single‐slice scanners' tube cooling considerations. Therefore, comparable protocols were established (see Table 3).

**Table 1:** Axial mode scan parameters for large‐scan FOV. Comparable scan parameters for the 85‐cm bore single‐slice and 70‐cm bore multislice scanners are indicated. Four scanning techniques were tested for each scanner with the largest scan field of view (SFOV) available on each scanner selected. The largest SFOV available on the multislice unit was 50 cm, while the large‐bore single‐slice only offered a SFOV of 60 cm.

	85‐cm Bore single slice			70‐cm Bore multislice		
Scan type	mAs	Slice thickness (mm)	Focal spot size	mAs	Slice thickness (mm)	Focal spot size
case A	120	5	large	120	5	small
case B	120	2	small	120	1.25	small
case C	400	5	large	400	5	large
case D	400	2	small	400	1.25	large

**Table 2:** Comparable scan parameters for the 70‐cm bore single‐slice and the 70‐cm bore multislice scanners are indicated. Four scanning techniques were tested for each scanner with the smallest scan field of view (SFOV).

	70‐cm Bore single slice			70‐cm Bore multislice		
Scan type	mAs	Slice thickness (mm)	Focal spot size	mAs	Slice thickness (mm)	Focal spot size
case E	125	5	small	120	5	small
case F	125	1.5	small	120	1.25	small
case G	400	5	large	400	5	large
case H	400	1.5	small	400	1.25	large

**Table 3:** Scanning protocols and parameters for each scanner are indicated. Identical scan parameters were not always possible; however, efforts were made to select comparable scanning techniques. The 70‐cm multislice and the 85‐cm single‐slice scanners were compared for three different scanning protocols (I, II, and III). The 70‐cm single slice scanner was compared to the large bore and the multislice scanners for protocol II. Both the GE (Hu) and McCollough (or IECS) definitions of pitch are included. For the multislice unit, the HQ mode (pitch = 3) was selected for all scanning protocols. For this scanner, slice thicknesses not consistent with the indicated pitch values were generated postscanning using z‐filtering or a variable thickness helical reconstruction algorithm, which is designed to control the tradeoff of the slice thickness versus image noise and artifacts.

Scanner: Protocol	mA	Pitch (GE definition)	Pitch (IECS definition)	Slice thickness (mm)	Table speed (mm/rot)
85‐cm bore single slice: I	250	1.5	1.5	3	4.5
70‐cm bore multislice: I	280	3	0.75	2.5	3.75
85‐cm bore single slice: II	250	2	2	3	6
70‐cm bore multislice: II	280	3	0.75	1.25	3.75
70‐cm bore single slice: II	250	2	2	3	6
85‐cm bore single slice: III	200	1.3	1.3	5	6.5
70‐cm bore multislice: III	200	3	0.75	5	7.5
70‐cm bore multislice: IV	380	3	0.75	2.5	7.5

Focal spot size is another important parameter that has some effect on image quality, in particular, the spatial resolution of the system. Focal spot size is selected based on the scanning technique employed. The 85‐cm single‐slice unit automatically selects the focal spot size based on slice thickness, with a small focal spot selected for a slice thickness of 2 mm and a large focal spot selected for all other larger slice thicknesses. The 70‐cm bore multislice unit, on the other hand, automatically selects a focal spot size based on power requirements, with a small focal spot size selected for a scanning technique less than 24 kW (e.g., 10 mA to 200 mA at 120 kV). The focal spot size on the 70‐cm bore single‐slice scanner is selected by the operator but is limited to specific scanning parameters. As a result, comparable focal spot sizes for each scanner was not possible for some single‐slice helical mode scanning protocols.

The same or similar user‐adjusted data and image‐processing parameters, DFOVs, reconstruction algorithms, and matrix sizes were also selected for each scanner. The DFOV selected for all scanners was 250 mm with a display matrix of 512×512 pixels. A standard reconstruction algorithm was also selected.

### A. Noise determination

Image noise was evaluated using a uniformity module contained in the Catphan® phantom, cast with a uniform material designed to be within 2% of the density of water. Image noise is expressed as a standard deviation of Hounsfield units. The average standard deviation was calculated from standard deviation measurements made from four adjacent slices, each contained within the uniformity module. The user‐defined region of interest from which a standard deviation was determined was consistent in area and location for each slice and each scanner. Comparisons of the average standard deviation were made for each scanner and each scanning protocol.

### B. Spatial frequency limits

Image noise and blurring place upper limits on the spatial frequencies of the patient/phantom reproduced in the image. At low contrasts, object visualization is limited by image noise. At high contrasts, object visualization is limited only by blurring sources.^(^ ^6^ ^)^ Two tests are used to evaluate the spatial frequency limits, including LCD tests to evaluate the effect of noise on perceptibility limits, and a high‐contrast resolution test using either the MTF or a high spatial frequency pattern for evaluation of blurring factors.^(^ ^5^ ^,^ ^6^ ^)^

### C. Low‐contrast detectability

The Catphan® phantom was equipped with an LCD module having three nominal contrast levels of 1.0%, 0.5%, and 0.3%. At each contrast level, there were nine supra‐slice targets with diameters of 15 mm, 12 mm, 9 mm, 7 mm, 5 mm, 4 mm, 3 mm, 2 mm, and 1 mm. These supra‐slice low‐contrast targets were cylinders spanning 40 mm, a length sufficient to prevent partial volume averaging (although phantom misalignment can induce such effects) and arranged in radial symmetric patterns to minimize nonuniformity effects of the CT scanner. LCD is reported as the number of detectable objects at each contrast level. For example, five objects implies that the five largest targets were detectable. The average number of objects detectable for each contrast level was determined from three adjacent image slices, each contained within the LCD module. The minimum resolvable target is also reported. The image window width and level were adjusted to provide maximum visibility of all test objects. Window widths and levels were comparable for each scanner.

### D. Modulation transfer function

Frequency domain analysis of the image resolution was determined using the MTF. The MTF was calculated using a 0.28‐mm diameter tungsten carbide bead cast in a uniform material in the Catphan® phantom. A profile density through the center of the bead was obtained on the image plane. This corresponds to the point spread function (PSF). A line spread function (LSF) was then derived from the PSF profile. MATLAB was then used to calculate the MTF using a fast Fourier transform of the LSF data.

In order to determine an accurate PSF not limited by the size of the display pixel, a retrospective reconstruction of the raw data was performed at a DFOV of 100 mm. This was the smallest DFOV that could be selected on the 70‐cm bore multislice unit that was also available on the other two scanners. This 100‐mm DFOV in conjunction with the 512×512 display matrix characteristic of each scanner minimized the potential for loss of signal data due to inadequate resolution. After the retrospective reconstruction of the raw data, a region of interest including and surrounding the bead was drawn, from which a 2D array of the CT values arising from the impulse source was generated. Following the Catphan® manual, the LSF was obtained by summing the CT numbers in each column of the 2D PSF array. Using a MATLAB script developed in‐house, a fast Fourier transform of the LSF data was performed, subsequently yielding the MTF. The MTF data of each scanner normalized to 1 at zero spatial frequency are reported at 50%, 10%, and 5% signal modulation for each scanning protocol.

### E. Slice thickness accuracy

Slice thickness accuracy was determined using the full width at half‐maximum (FWHM) pixel intensity technique. The Catphan® phantom was equipped with a module containing two sets of ramp wires angled at 23°, which were used to determine scan thickness by radiographically determining the length of the wire when the window width was set to 1 and the window level was set to the half‐maximum CT number of the ramp wire. This window level was determined by first identifying the CT number of the background when the window width was set to 1 and the level was adjusted to a point where the ramp almost disappeared. The CT number of the level at this position is the maximum value. The CT number corresponding to the background was then subtracted from the maximum CT number to establish a range, which was then multiplied by 0.5. This half‐range value was added to the background, and the resulting CT number corresponding to the half maximum was determined. The level was then set to this half maximum CT number, and the length of the ramp was measured using the tools provided by the scanner's software. In order to compensate for the angularity of the ramp in the phantom module, the ramp length measured was then multiplied by tan(23°). Four ramp length measurements were used to calculate an average ramp length. The error associated with the ramp length as compared to the indicated slice thickness was calculated, that is, the variance of the measured slice thickness from the reported slice thickness. Slice thickness accuracy was determined for both axial and helical mode scanning protocols.

### F. Slice sensitivity profile: Helical CT

Effective slice thickness was also evaluated with the slice sensitivity profile (SSP), which is defined as the system PSF restricted to the line along the direction of table motion and through the center of the gantry aperture. It also describes the longitudinal *(z*‐axis) resolution. According to a method described in the Catphan® manual, the SSP was determined for helical mode scanning by incrementing a slice through a tungsten carbide bead and reconstructing images in positive and negative *z* directions from the bead and plotting the peak CT number of the bead image in each slice. Images were reconstructed at 0.5‐mm intervals. This was the smallest increment that could be selected on each scanner without causing processing problems due to computational limitations. The maximum CT number of the bead on each slice was determined, as was the CT number corresponding to the background. The CT numbers were plotted as a function of distance from the impulse bead source, generating a slice sensitivity profile. Using the CT number of the background and the maximum CT number of the bead, a range was determined and then multiplied by 0.5. The value corresponding to half of the range was then added to the background CT number, the result of which is referred to as the half maximum CT number. The profile width corresponding to this half maximum CT number (or FWHM) was measured. The length of the bead at the FWHM pixel intensity indicates the SSP.

### G. CTDI

Computer tomography dose index was also measured according to procedures reported in the literature.^(^ ^5^ ^–^ ^4^ ^,^ ^11^ ^)^ Pencil CT ionization chambers were used to calculate CTDI in specialized head and body phantoms. The same CTDI exposure and measurement techniques were used for each scanner.

## IV. RESULTS AND DISCUSSION

### A. Axial mode

#### A.1 Noise

Random variation in pixel numbers about some mean value is image noise and is comprised of electronic noise and quantum noise, the latter component dominating in clinical situations. Quantum noise arises from the statistical uncertainty in the finite number of transmitted X‐ray photons. Those factors that reduce the number of transmitted photons in a region of interest, such as decreasing slice thickness, lead to larger variations in pixel numbers and therefore increased image noise. The results showed that both the 70‐cm bore multislice unit (500 mm SFOV) and the 85‐cm bore single‐slice unit (600 mm SFOV) responded as predicted, that is, as slice thickness decreases and milliamperes increase, the noise levels increase and decrease, respectively. Still, the noise levels of the 70‐cm bore multislice unit were consistently lower than those of the 85‐cm bore single slice unit for each scanning protocol. In addition, the relative changes in noise content with respect to changing slice thickness and photon intensity differ significantly for each scanner. The increase in noise content as a function of decreasing slice thickness was evaluated. At a lower exposure level (120 mAs), a 60% reduction in slice thickness resulted in a 34% increase in noise with the 85‐cm bore single slice unit, while the 70‐cm bore multislice unit indicated a much more drastic increase in noise content (119% increase) for a 75% reduction in slice thickness. At 400 mAs, the 70‐cm bore multislice unit demonstrated the same relative increase in noise levels for an equivalent slice thickness reduction, while the 85‐cm bore single slice unit indicated a nonintuitive 64% increase in noise for a 60% decrease in slice thickness, despite the higher exposure levels. For constant slice thickness and increasing exposure levels (120 mAs to 400 mAs), noise levels decreased by about 45% for both scanners with a 5‐mm slice thickness, and as well for the 70‐cm bore multislice unit at a 1.25‐mm slice thickness. With a 2‐mm slice thickness, a 33% reduction in noise was observed with the 85‐cm single slice unit when increasing exposure level from 120 mAs to 400 mAs.

The noise levels of the 70‐cm bore multislice unit were consistently and significantly lower than those of the 70‐cm single slice unit for each scanning protocol. The increase in noise content as a function of decreasing slice thickness was evaluated for each scanner. At low exposure levels (120 mAs to 125 mAs), a 75% reduction in slice thickness on the 70‐cm bore multislice unit and a 70% slice thickness reduction on the 70‐cm bore single‐slice unit resulted in noise level increases of 118% and 52%, respectively. At much higher exposure levels (400 mAs) and for the same relative reduction in slice thickness, noise levels increased by approximately 112% and 83% on the 70‐cm bore multislice unit and the 70‐cm bore single slice unit, respectively. For constant slice thickness (5 mm) and increasing exposure levels (120 mAs to 400 mAs), noise levels decreased by about 42% to 45% for both scanners. For a 1.25‐mm slice thickness, noise content decreased by 46.5% on the 70‐cm bore multislice unit. For a 1.5‐mm slice thickness on the 70‐cm bore single slice unit, a 30% noise reduction was observed from 125 mAs to 400 mAs.

#### A.2 Low‐contrast detectability

**Figure 1:**
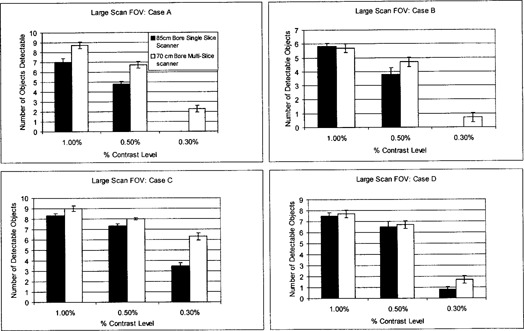
Low‐contrast detectability (LCD) bar graph for axial mode scanning: large‐scan FOV

**Figure 2:**
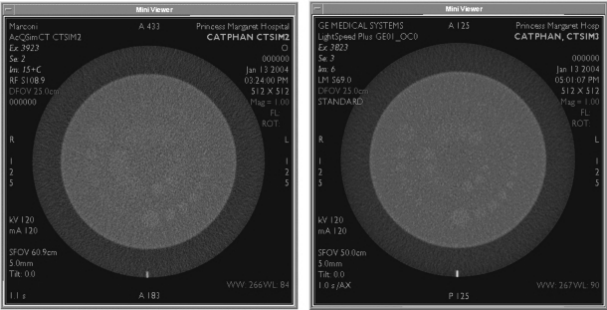
Low‐contrast detectability (LCD): large‐scan FOV. LCD was evaluated for the 85‐cm bore single‐slice scanner (left) and the 70‐cm bore multislice scanner (right) using the Catphan® phantom's LCD module consisting of low‐contrast targets of nominal contrast levels of 1.0%, 0.5%, and 0.3%. An axial scan was performed with a 5‐mm slice thickness, 120 mAs, DFOV of 25 cm, and a standard reconstruction algorithm (case A). The 70‐cm multislice scanner shows better LCD, particularly for very low‐contrast targets of 0.3%.

For a large‐scan FOV, Fig. 1 shows that both scanners have similar degrees of LCD at 1.0% and 0.5% nominal contrast levels. Low‐contrast targets of 0.3% are detectable with the 70‐cm bore multislice scanner for all scanning protocols, whereas detectability of this low‐contrast target with the 85‐cm bore single‐slice unit is only possible at 400 mAs. The minimum resolvable low‐contrast targets for the suboptimal low‐contrast scanning parameters of scanning protocol B (i.e., lowest mAs and smallest slice thickness) are 15 mm at 0.3% and 7 mm at 0.5% for the 70‐cm bore multislice and 85‐cm bore single‐slice units, respectively (see Fig. 2).

**Figure 3:**
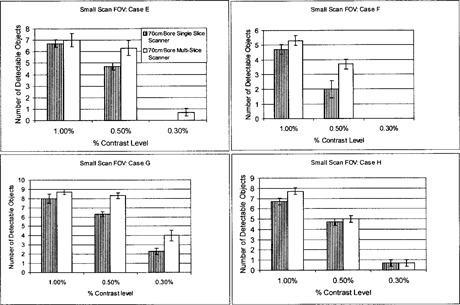
Low‐contrast detectability (LCD) bar graph for axial mode scanning: small‐scan FOV

**Figure 4:**
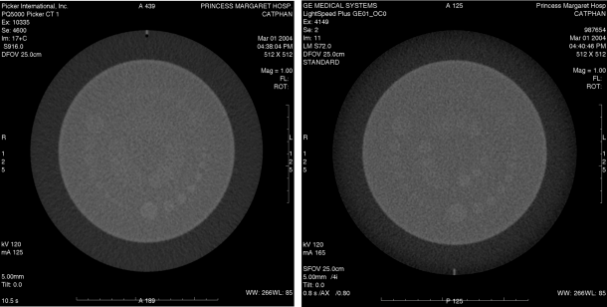
Low‐contrast detectability (LCD): small‐scan FOV. LCD was evaluated for the 70‐cm bore single‐slice scanner (left) and the 70‐cm bore multislice scanner (right) using the Catphan® phantom's LCD module consisting of low‐contrast targets of nominal contrast levels of 1.0%, 0.5%, and 0.3%. An axial scan was performed with a 5‐mm slice thickness, 132 mAs (165mA×0.8s) on the multislice scanner and 132 mAs (125mA×1.05s) on the single‐slice scanner with DFOVs of 25 cm and a standard reconstruction algorithm (case E).

For a small‐scan FOV, Fig. 3 shows that both scanners have similar degrees of LCD at the 1.0% contrast level. At lower‐contrast levels of 0.5% and 0.3%, the 70‐cm bore multislice unit was superior. The 70‐cm bore single‐slice unit is unable to adequately resolve targets at a 0.3% contrast level for lower exposure levels. The minimum resolvable low‐contrast targets at the suboptimal LCD parameters of scanning protocol F (i.e., lowest exposure level and smallest slice thickness) are 9 mm at 0.5% and 12 mm at 0.5% for the 70‐cm bore multislice and single‐slice units, respectively (see Fig. 4).

The multislice scanner demonstrated superior LCD in comparison to the large 85‐cm bore and standard 70‐cm bore single‐slice scanners, particularly at the very low‐contrast levels of 0.3%. This can be attributed in great part to the lower noise levels of the multislice scanner because image noise has a spatial frequency content limiting the perceptibility of low‐contrast detail.^(^ ^5^ ^,^ ^6^ ^)^

#### A.3 Modulation transfer function

Limiting spatial resolution of an imaging system is specified at the 5% MTF level. An acceptable limit is generally specified to be greater than 5lp/cm (1 mm).^(^ ^12^ ^)^ The limiting spatial resolution of each scanner exceeded the acceptable limit of 5lp/cm (Tables 4 and 5). The results obtained for each scanner were consistent with predicted trends, that is, as slice thickness decreased, the MTF improved. This can be attributed to the increased collimation required for thinner slices, which reduces the amount of scattered photons that ultimately contribute to the “blurriness” of the image. With less “blurring,” there is less spreading of the impulse bead point source used to obtain the MTF, and the quality/gain of the high‐frequency signal is somewhat improved. For the 70‐cm multislice unit, however, the quality of the signal at higher frequencies, while clinically acceptable, was poorer in comparison to both single‐slice scanners. This may be attributed to the focal spot size of the multislice scanner, which when too large, can limit spatial resolution. Spatial resolution is also influenced by ray spacing within a projection data set. Ray spacing must overlap to prevent aliasing. A reduction in sample spacing will improve resolution but is limited in third‐generation scanners (the tube and the detector array are attached to the same rotating frame and move simultaneously) by detector spacing.^(^ ^6^ ^)^ The multislice unit is a third‐generation scanner, whereas the single‐slice units are both fourth‐generation scanners (X‐ray tube rotates about fixed detectors positioned 360°).

**Table 4:** Modulation transfer function (MTF) for axial mode scanning: large SFOV. The results are reported at the 50%, 10%, and 5% MTF.

	50 % MTF		10% MTF		5% MTF	
Scan type	85‐cm Bore single slice	70‐cm Bore multislice	85‐cm Bore single slice	70‐cm Bore multislice	85‐cm Bore single slice	70‐cm Bore multislice
case A	3.6	3.2	8.2	4.9	9.2	5.3
case B	3.6	3.6	7.8	5.8	8.8	6.4
case C	3.5	3.3	7.1	4.9	8.1	6.1
case D	3.8	3.8	7.4	6.2	8.2	6.6

**Table 5:** Modulation transfer function (MTF) for axial mode scanning: small SFOV. The results are reported at the 50%, 10%, and 5% MTF.

	50 % MTF		10% MTF		5% MTF	
Scan type	70‐cm Bore single slice	70‐cm Bore multislice	70‐cm Bore single slice	70‐cm Bore multislice	70‐cm Bore single slice	70‐cm Bore multislice
case E	5.3	3.5	9.3	5.3	10.1	5.7
case F	6.1	4.0	11.1	6.3	13.0	7.1
case G	6.0	3.3	9.6	5.1	10.8	5.4
case H	7.5	3.8	12.5	6.4	13.5	7.0

#### A.4 Slice thickness accuracy

**Figure 5:**
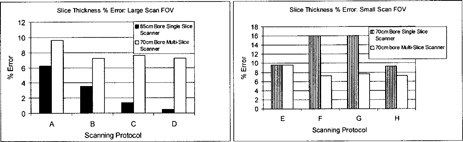
Slice thickness accuracy for axial mode scanning. The accuracy of the slice thickness is reported for both large‐ and small‐scan FOVs. The Catphan® module containing angled wire ramps was used to measure slice thickness. The error of the measured slice thicknesses is reported.

Figure 5 shows that all measured FWHM ramp lengths of all three scanners are within ±0.5mm of the scanner indicated slice thickness. All the results generally indicated good precision. At the large‐scan FOV, the 85‐cm bore single‐slice scanner showed better accuracy as compared to the slice thickness measurements of the multislice scanner. At the small‐scan FOV, the multislice scanner slice thickness measurements showed better accuracy as compared to that of the 70‐cm bore single‐slice scanner.

#### A. 5 CTDI

Table 6 shows CTDI measured for each scanner as well as the manufacturers' reported data.^(^ ^11^ ^)^ The data on multislice agree well with McCollough.^(^ ^3^ ^)^ Measured values were similar on all three scanners and are within the acceptable range of industry standards. It is also interesting to note that CTDI values on the multislice were slightly higher than those of both single‐slice scanners. According to AAPM No. 74, that can be attributed to the slightly larger radiation profiles required to exclude beam penumbra from the outer detectors.^(^ ^6^ ^)^

**Table 6:** CTDI: Axial mode scanning. Center and surface doses (CTDI, mGy), f‐factor for acrylic=0.78(CGy/R). Measurements on axial mode, 120 kVp, 200 mAs and a nominal scan width of 10 mm.

Phantom portion	85‐cm Single slice	70‐cm Single slice	70‐cm multislice	Industry data
head: center	28.2	27	39.2	32‐73
head: surface	34.0	25.6	39	32‐76
body: center	8.4	9	10.9	11‐20
body: surface	20.16	21.6	22.9	20‐42

### B. Helical mode

#### B.1 Noise

The multislice unit shows lower noise levels than both single‐slice scanners for helical scanning protocols I and III (Table 7), which is generally consistent with the results generated for axial mode scanning. For scanning protocol II, noise levels are comparable for all three scanners, which may be attributed to the small slice thickness of 1.25 mm implemented for this scanning protocol.

**Table 7:** A uniformity module contained in the Catphan® phantom was used to evaluate noise levels. The results are reported as standard deviations of Hounsfield numbers. Note that only one protocol for the 70‐cm single‐slice scanner was evaluated.

Scan type	85‐cm Single slice	70‐cm Multislice	70‐cm Single slice
I	7.4	5.1	_—_
II	7.1	7.6	6.4
III	9.9	4.1	—
IV	—	4.5	—

#### B.2 Low‐contrast detectability

**Figure 6:**
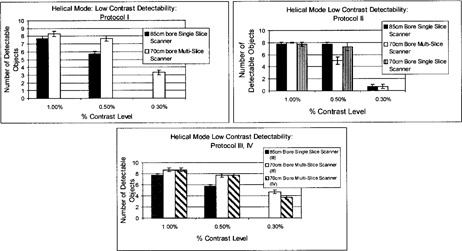
Low‐contrast detectability (LCD) bar graph for helical scanning mode: protocols I, II, III, and IV

Detectability of the 1.0% and 0.5% low‐contrast targets is generally comparable for the 85‐cm bore single‐slice and 70‐cm bore multislice units, although the latter system indicates slightly better results (Fig. 6). At a 0.3% nominal contrast level, the multislice scanner demonstrates superior LCD as compared to the large‐bore single‐slice unit. The minimum resolvable low‐contrast target for all scanning protocols is 5 mm at 0.3% and 15 mm at 0.3% for the multislice and 85 cm large‐bore single‐slice units, respectively. The multislice unit demonstrates superior LCD in comparison to the large‐bore and standard‐bore, single‐slice scanners, particularly at the very low‐contrast levels of 0.3%. Evidently, the reduced noise levels of the multislice scanner are reflected in the ability to resolve small, low‐contrast targets. These results are consistent with those generated under axial scanning mode protocols.

#### B.3 Modulation transfer function

Generally, the MTF results generated for both the 85‐cm bore single‐slice and the 70‐cm bore multislice scanners are comparable for most of the scanning protocols (Table 8). Still, the multislice scanner shows increased signal degradation as compared to the larger‐bore single‐slice unit, which is consistent with the MTF results generated for axial mode scanning. The 70‐cm bore single‐slice scanner showed relatively poorer limiting spatial resolution for helical mode scanning, as compared to the other two scanning systems.

**Table 8:** Modulation transfer function (MTF) for helical mode scanning. The results are reported at the 50%, 10%, and 5% MTF.

	50% MTF			10% MTF			5% MTF		
Scan type	85‐cm Single slice	70‐cm Multislice	70‐cm Single slice	85‐cm Single slice	70‐cm Multislice	70‐cm Single slice	85‐cm Single slice	70‐cm Multislice	70‐cm Single slice
I	3.6	4.3	—	7.7	7.7	—	8.8	8.4	—
II	3.4	3.8	3.0	7.5	6.1	4.7	8.8	7.0	5.1
III	3.3	3.3	—	8.5	5.3	—	9.0	6.4	—
IV	—	3.8	—	—	6.5	—	—	7.8	—

#### B.4 Slice thickness verification: Ramp method and SSP method

**Figure 7:**
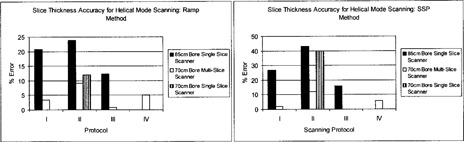
Slice thickness accuracy for helical mode scanning. The accuracy of the slice thickness is reported for all scanning protocols. The Catphan® module containing angled wire ramps was used to measure slice thickness. Slice thickness was also evaluated using SSP generated from incrementing axial slices through a bead in the longitudinal direction. The error of the measured slice thicknesses is reported.

The FWHM ramp lengths measured for all three scanners are within ±0.75mm of the scanner indicated slice thickness (Fig. 7); however, the slice thickness accuracy of the multislice scanner is superior. In addition, helical mode scanning yields poorer slice thickness accuracy for both single‐slice scanners, as compared to axial mode scanning. This can be attributed to the table motion and subsequent interpolation process characteristic of helical CT, which results in broadening of the response function of the detector array.^(^ ^13^ ^)^ In spite of the broadened response function characteristic of helical CT, however, Wang and Vannier^(^ ^13^ ^)^ indicate that this scanning mode allows for improved longitudinal resolution due to the retrospective reconstruction capabilities of the scanner, particularly reconstructions at slice thicknesses smaller than the scan slice thickness. This is particularly important for applications requiring high‐longitudinal resolution, for example, lesion detection and measurement. In the case of the multislice scanner, slice thickness accuracy was comparable for both axial and helical mode scanning. This may be attributed to a pitch of 0.75 (IECS pitch definition), which due to the scanning overlap, results in a larger number of data points from which the slices are generated, improving the slice thickness and longitudinal resolution accuracy.

Slice sensitivity profiles from the multislice scanner also showed better slice thickness accuracy, as compared to the other two single‐slice scanners. These results, while they are not directly comparable, are consistent with those theoretically demonstrated by Liang and Kruger,^(^ ^14^ ^)^ who showed a dual‐slice scanner to have better longitudinal resolution (SSP) than the single‐slice scanner to which it was compared. For a given scan rate and the same scanned volume, multiple detector rows (*N* rows) generate *N* times the number of projections and improve the resolution in the longitudinal direction, as compared to a single‐slice (one row of detectors) scanner. It is also interesting to note that the error associated with the SSP‐determined slice thickness is generally greater than the error associated with the ramp‐determined slice thickness. This may be due to the actual, physical variance in the slice thickness. The SSP is generated from many slices, which are assumed to be of equal size. In practice, however, they may not have the same size. The variation of each slice from the ideal thickness is subsequently reflected in the SSP. Therefore, use of the SSP for slice thickness determination may not be the most reliable method.

## V. CONCLUSIONS

The objective of this study was to evaluate and compare the image quality of three different CT simulation scanners used for radiation treatment planning at Princess Margaret Hospital. We evaluated image quality in terms of noise levels, LCD, limiting spatial resolution (MTF), and slice thickness accuracy, according to guidelines set out by AAPM No. 39. For axial mode scanning, the overall image quality of each scanner was generally comparable; however, the multislice scanner was superior in terms of image noise and LCD, while the single‐slice scanners proved better in terms of limiting spatial resolution. Garcia‐Ramirez et al.^(^ ^15^ ^)^ also reported comparable image quality performance between a large 85‐cm bore single‐slice unit and a standard 70‐cm bore size single‐slice unit, when evaluated for axial mode scanning.

For helical mode scanning, the overall image quality of each scanner was also comparable. Still, the multislice scanner demonstrated superior performance in terms of image noise. The clinical impact of this improvement in noise content was most apparent in the detectability of very low‐contrast objects, specifically, very small objects with a nominal low‐contrast level of 0.3%, which was not achievable with the other two scanners. Additional postprocessing filters, generally necessary for the delineation of low‐contrast soft tissue targets, may not be required due to the improved detection of low‐contrast objects. Limiting spatial resolution, slice thickness accuracy, and longitudinal resolution were comparable for each scanner. In this study, a pitch of 3:1 (0.75 IECS defined pitch) was selected on the multislice scanner. This implies overlapping acquisition, which is generally used to improve image quality while somewhat compromising volume coverage speed. The other available pitch selection on this scanner intended for fast volume coverage speed was not selected, since the objective of this study was simply to compare image quality as opposed to scanning time. It is worth pointing out, however, that a recent study by Sahani et al.^(^ ^16^ ^)^ showed no statistically significant differences in the overall image quality performance of the 3:1 or 6:1 pitch imaging techniques. However, side‐by‐side comparisons of images generated by both pitch selections showed superior quality with the images generated using a pitch of 3:1.

Drastic improvements in image quality were not apparent with the multislice scanner except in the areas of image noise and LCD. However, a multislice scanner is a critical tool for an RT planning facility. Multislice scanning dramatically improves volume coverage speed without compromising image quality performance and without increasing radiation dose to the patient. High‐longitudinal (*z*‐axis) resolution is achieved and scanning time is reduced. This minimizes image artifacts due to sudden patient motion, which for radiation treatment planning is a significant consideration, particularly for pediatric patients.

Multislice scanning is also particularly useful for dynamic CT imaging. For example, breathing motion is a significant source of error in RT planning for the thorax and upper abdomen, causing artifacts and distortions in treatment planning CT scans acquired, which can lead to significant dose delivery errors. Traditionally, breath‐hold techniques have been employed to reduce artifacts due to respiratory motion but are limited by the poor breath‐hold tolerance of lung cancer patients. Multislice scanning can be exploited to account for the breathing motion in RT treatment planning. Multislice scanners, when operated in cine mode, enable the simultaneous collection of multiple CT scans with digital‐spirometry over many free breathing cycles to create a 4D image set, where tidal lung volume is the additional dimension. The collection of volumetric patient data over time (not possible with single‐slice scanners) can then be used to model tumor and lung motion during breathing or to determine the extent of tumor motion for margin determination in the treatment‐planning process.^(^ ^17^ ^)^ Cardiac imaging has also benefited from the capabilities of multislice scanning, which has been used to measure coronary motion, which is then correlated with cardiac image quality. In fact, ECG‐gated spiral CT using multislice scanners has made it possible to scan the entire heart in a single breath hold, while continuous data acquisition makes it possible to reconstruct the multiple phases of the cardiac cycle.^(^ ^18^ ^)^

## ACKNOWLEDGMENTS

We would like to thank Biu Chan, a radiation therapist at Princess Margaret Hospital, for his tremendous technical support and guidance.
